# Application and underlying mechanism of acupuncture for the nerve repair after peripheral nerve injury: remodeling of nerve system

**DOI:** 10.3389/fncel.2023.1253438

**Published:** 2023-10-24

**Authors:** Yongke Yang, Chang Rao, Tianlong Yin, Shaokang Wang, Huiyan Shi, Xin Yan, Lili Zhang, Xianggang Meng, Wenlong Gu, Yuzheng Du, Feng Hong

**Affiliations:** ^1^Beilun District People’s Hospital, Ningbo, China; ^2^Tianjin Union Medical Center, Tianjin, China; ^3^First Teaching Hospital of Tianjin University of Traditional Chinese Medicine, Tianjin, China; ^4^National Clinical Research Center for Chinese Medicine Acupuncture and Moxibustion, Tianjin, China; ^5^National Anti-Drug Laboratory Beijing Regional Center, Beijing, China

**Keywords:** acupuncture, peripheral nerve injury, mechanism, nerve system remodeling, nerve repair

## Abstract

Peripheral nerve injury (PNI) is a structural event with harmful consequences worldwide. Due to the limited intrinsic regenerative capacity of the peripheral nerve in adults, neural restoration after PNI is difficult. Neurological remodeling has a crucial effect on the repair of the form and function during the regeneration of the peripheral nerve after the peripheral nerve is injured. Several studies have demonstrated that acupuncture is effective for PNI-induced neurologic deficits, and the potential mechanisms responsible for its effects involve the nervous system remodeling in the process of nerve repair. Moreover, acupuncture promotes neural regeneration and axon sprouting by activating related neurotrophins retrograde transport, such as nerve growth factor (NGF), brain-derived neurotrophic factor (BDNF), glial cell-derived neurotrophic factor (GDNF), N-cadherin, and MicroRNAs. Peripheral nerve injury enhances the perceptual response of the central nervous system to pain, causing central sensitization and accelerating neuronal cell apoptosis. Together with this, the remodeling of synaptic transmission function would worsen pain discomfort. Neuroimaging studies have shown remodeling changes in both gray and white matter after peripheral nerve injury. Acupuncture not only reverses the poor remodeling of the nervous system but also stimulates the release of neurotrophic substances such as nerve growth factors in the nervous system to ameliorate pain and promote the regeneration and repair of nerve fibers. In conclusion, the neurological remodeling at the peripheral and central levels in the process of acupuncture treatment accelerates nerve regeneration and repair. These findings provide novel insights enabling the clinical application of acupuncture in the treatment of PNI.

## Graphical abstract

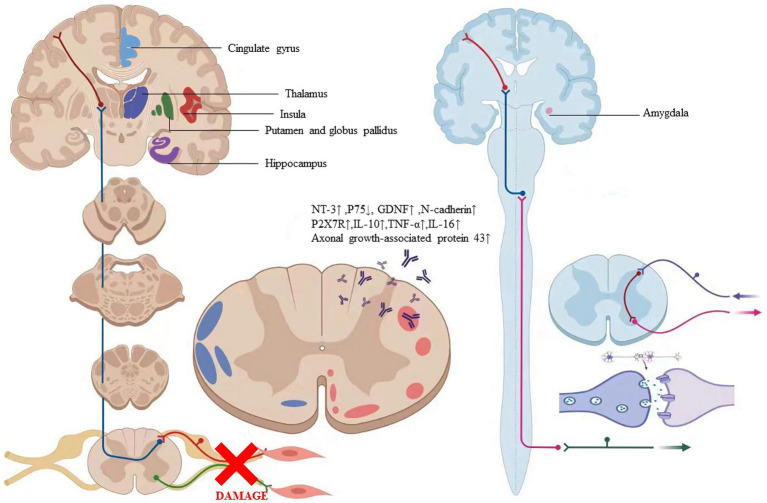

## 1. Introduction

Peripheral nerve injury caused by compression, traction, ischemia, and laceration can cause motor impairment of the limb, disuse atrophy of the limb muscles, and other nutritional disorders and sensory disturbances in the damaged innervated areas. With a 13–23 per 100,000 person-years estimated incidence rate, peripheral nerve damage (PNI) can cause sensory loss, persistent discomfort, motor impairment, or even amputation (Asplund et al., 2009; Sullivan et al., 2016). The most popular nerve injury categorization system is Seddon’s original one based on neurophysiological alterations: grade 1 nerve damage is a neurapraxia disease, grade 2 is axonal degeneration, and grade 3 is nerve transection. Peripheral nerve injury functional recovery is a slow process. The amount of time required for functional reconstruction and neural regeneration may be the reason for this, but the treatment method used also may be a factor in how long it takes to recover. Before the nerve injury becomes irreparable, early detection enables the start of neuroanastomosis, a suitable rehabilitation program, and adjustment of biomechanics, all of which benefit to encourage nerve repair. For instance, peripheral nerve injuries are more frequent in the upper extremities than the lower, tend to be sport-specific, and frequently include a biomechanical component. According to earlier research, people with peripheral nerve injury of the upper limbs did not have a high quality of life because of the acute neuropathic pain and potential for impairment (Pan et al., 2019). At present, there are gradually reconstructive operations clinically, but many patients experience a loss of their upper limbs’ normal function after the operation. Long-term nerve injury and inability to recover will also result in a lifetime impairment of patients. Therefore, further postoperative recovery is required to treat them (Bruyns et al., 2003; Magistroni et al., 2020). The main strategies for the treatment of PNI include pharmacologic interventions, behavioral therapy, physical stimulation, and supportive therapy in addition to surgery. In the numerous interventions for peripheral nerve injury, we need to carry out precision treatment according to the patient’s condition. Among them, acupuncture has its unique advantages in that acupuncture plays an important role in anti-inflammation, analgesia, and speeding up nerve repair. This makes the sequence of postoperative rehabilitation treatment of patients can be acupuncture first and rehabilitation manipulation later, to reduce the pain during rehabilitation manipulation and maximize the analgesic efficiency of acupuncture. Moreover, acupuncture is a safe, effective, and less painful treatment method. But there is still much uncertainty about how acupuncture works in PNI.

Acupuncture is a procedure involving the insertion of a fine needle into the skin or deeper tissues at specific locations of the body (acupoints) to prevent and treat diseases. Several lines of neuroanatomical and neurological evidence have demonstrated the abundant distribution of nerve endings in human meridians and acupoints, and the involvement of the nervous system is indispensable for the effects of acupuncture. Acupuncture is regarded as a potential adjuvant treatment for acupuncture analgesia, neural regeneration, and functional restoration in addition to conventional therapy of nerve anastomosis (Hao et al., 1995; Balogun et al., 1998; Zhang et al., 2018). Some studies showed that electroacupuncture (EA) helped individuals with peripheral nerve injuries improve their motor or sensory function (Dimitrova et al., 2017). Some researchers have indicated that rehabilitation training together with Jiaji electroacupuncture can greatly facilitate the recovery of muscle group function and improve the quality of life of patients with upper limb peripheral nerve injury (Li H. et al., 2021). With the rapid development of research on acupuncture’s central effect, accumulating evidence showed acupuncture or electro-acupuncture also played a key role in brain remodeling, which suggested a potentially positive impact on axon regeneration and synapse formation (Xiao et al., 2018). Some research suggests acupuncture and electroacupuncture can encourage nerve regeneration and enhance nerve function (Inoue et al., 2003; Lu et al., 2008; Chen et al., 2013). What’s more, acupuncture is green and harmless, which has this beauty, that accelerates the recovery after PNI.

Research on the mechanism of acupuncture in peripheral nerve injury is currently mostly concentrated at the level of individual molecules and/or signaling cascades. Acupuncture has the potential to control a complex network of several signaling molecules and pathways due to the wide range of interaction communication that exists between various signaling pathways. This notion is in line with acupuncture’s holistic regulation features, which involve numerous targets, linkages, techniques, and levels. And more research is needed to describe how acupuncture affects this intricate network. Therefore, this paper reviews the effects and mechanisms of acupuncture on nerve regeneration and repair from the perspective of nervous system remodeling.

## 2. Clinical efficacy of acupuncture or electro-acupuncture on nerve regeneration of PNI patients

**Figure 1:**
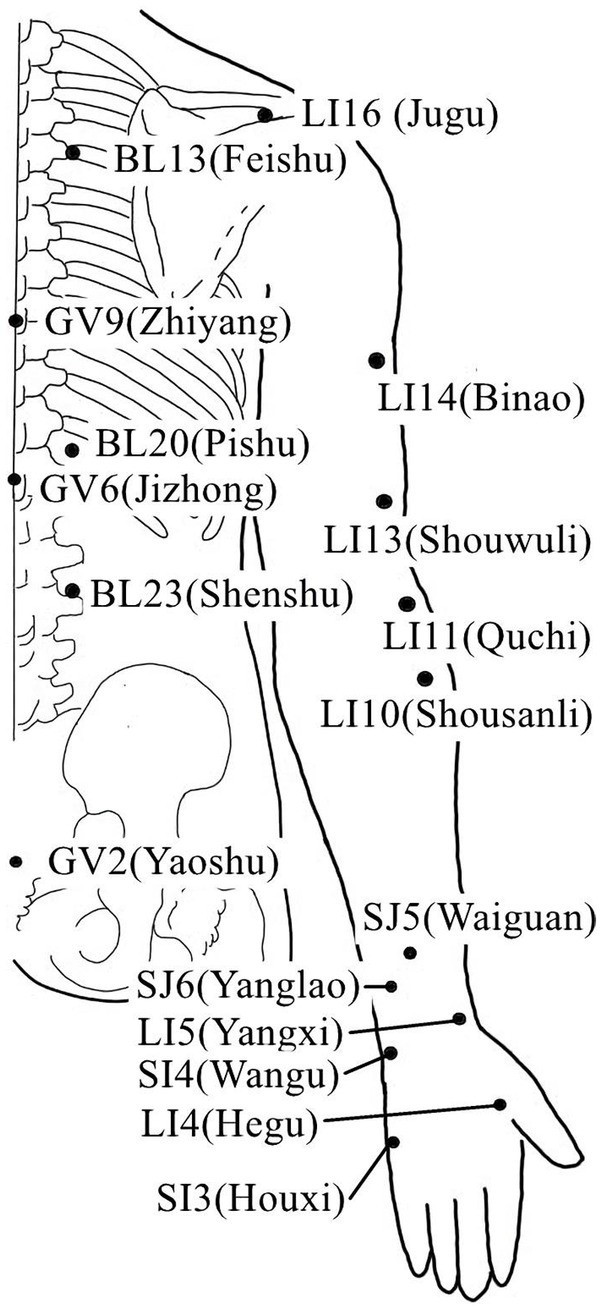
Acupoints on the upper limb lateral and back lumbar.

**Figure 2:**
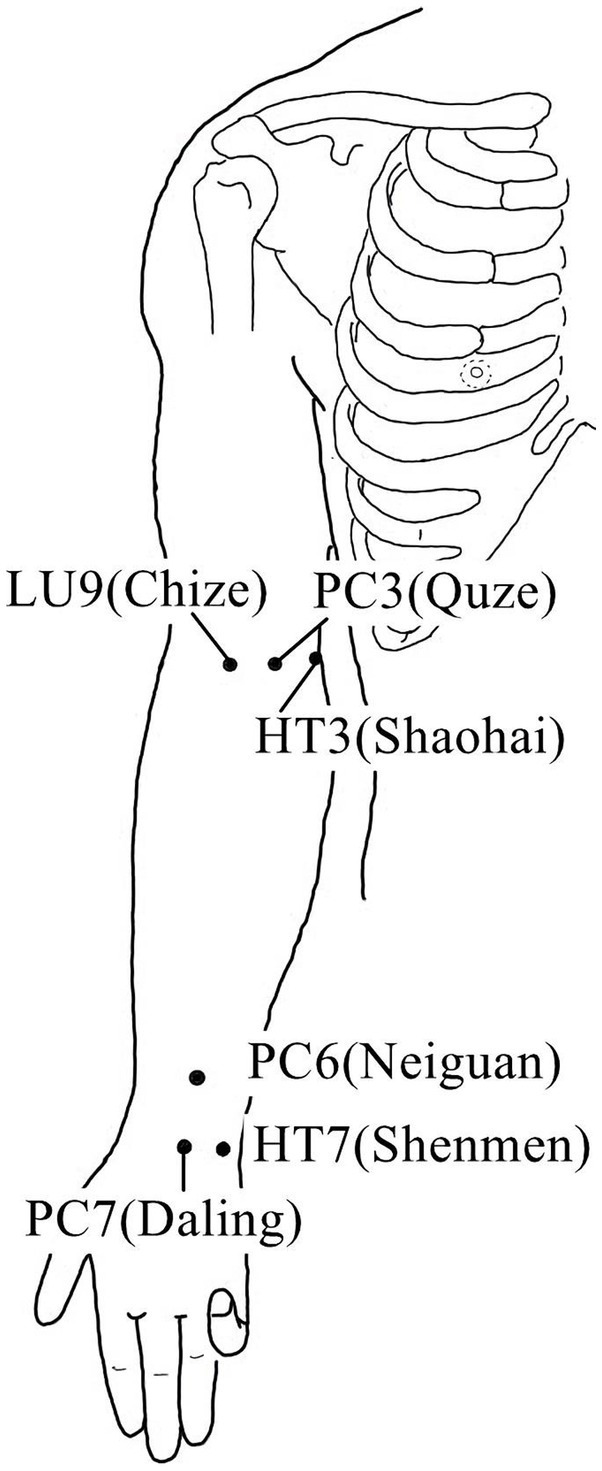
Acupoints on the inside of the upper limb.

**Figure 3:**
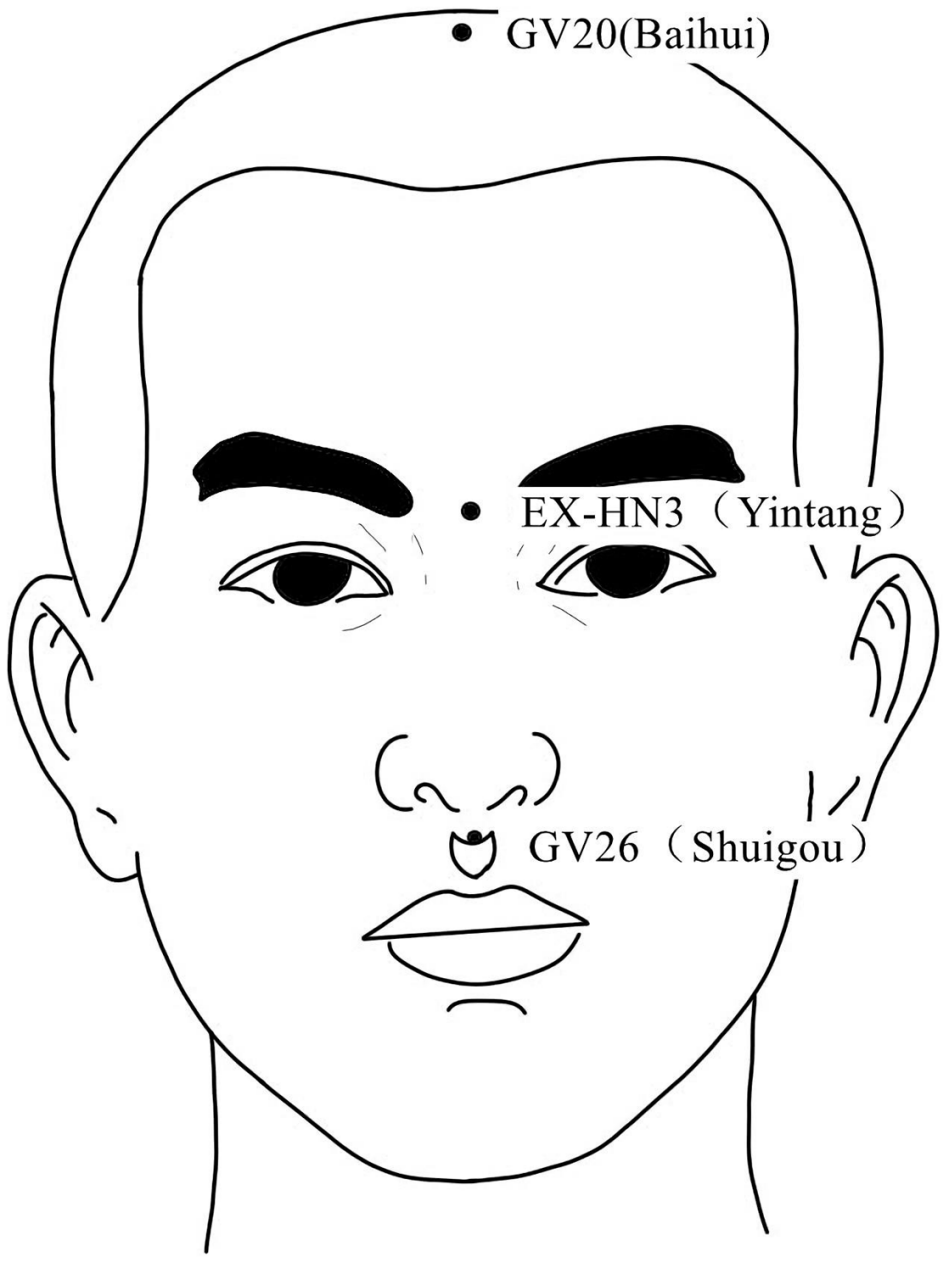
Acupoints on the head and face According to the newest National Standard of the People’s Republic of China GB/T 12346–2021 Nomenclature and Location of Meridia Points, Yintang belongs to Governor Vein (GV), in order to be consistent with the literature cited in this paper, Yintang is still labeled EX-HN3.

**Figure 4:**
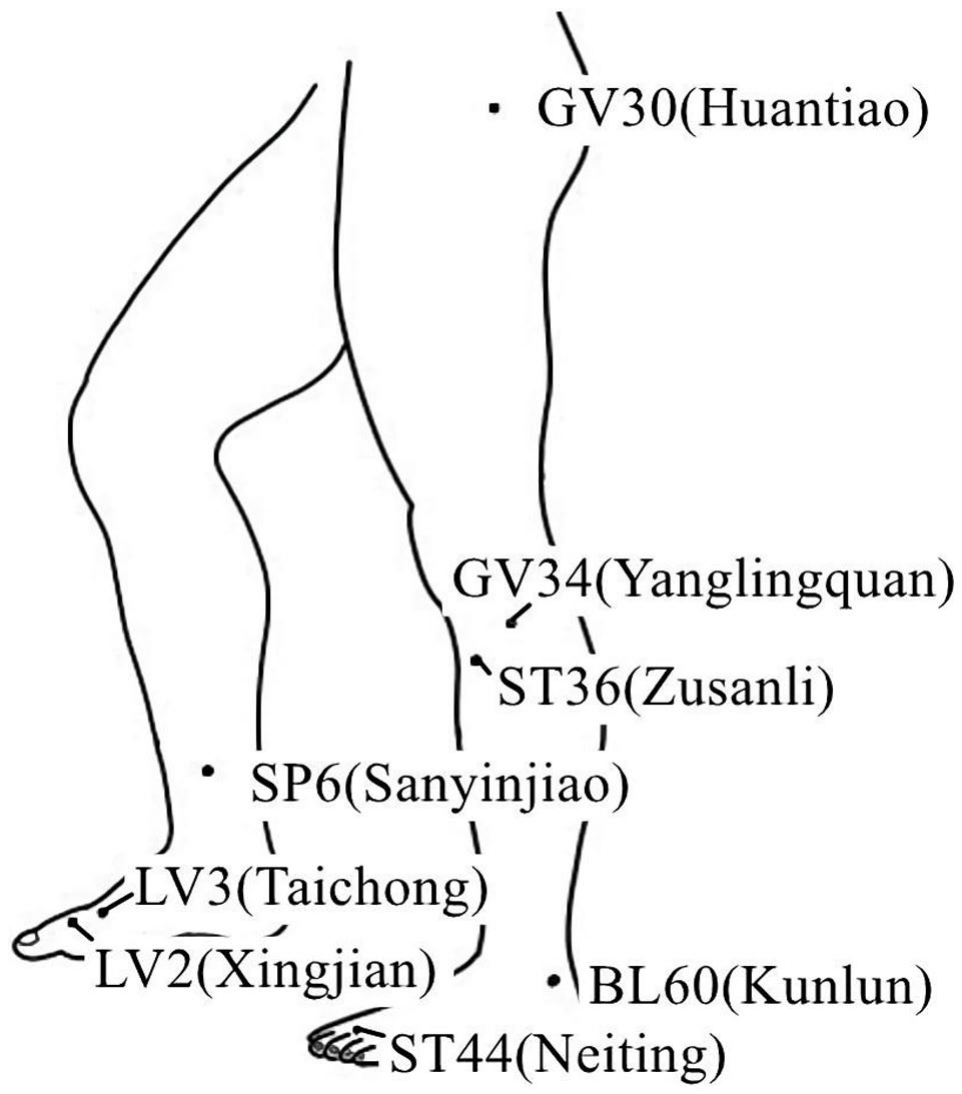
Acupoints on the lower extremity.

**Figure 5:**
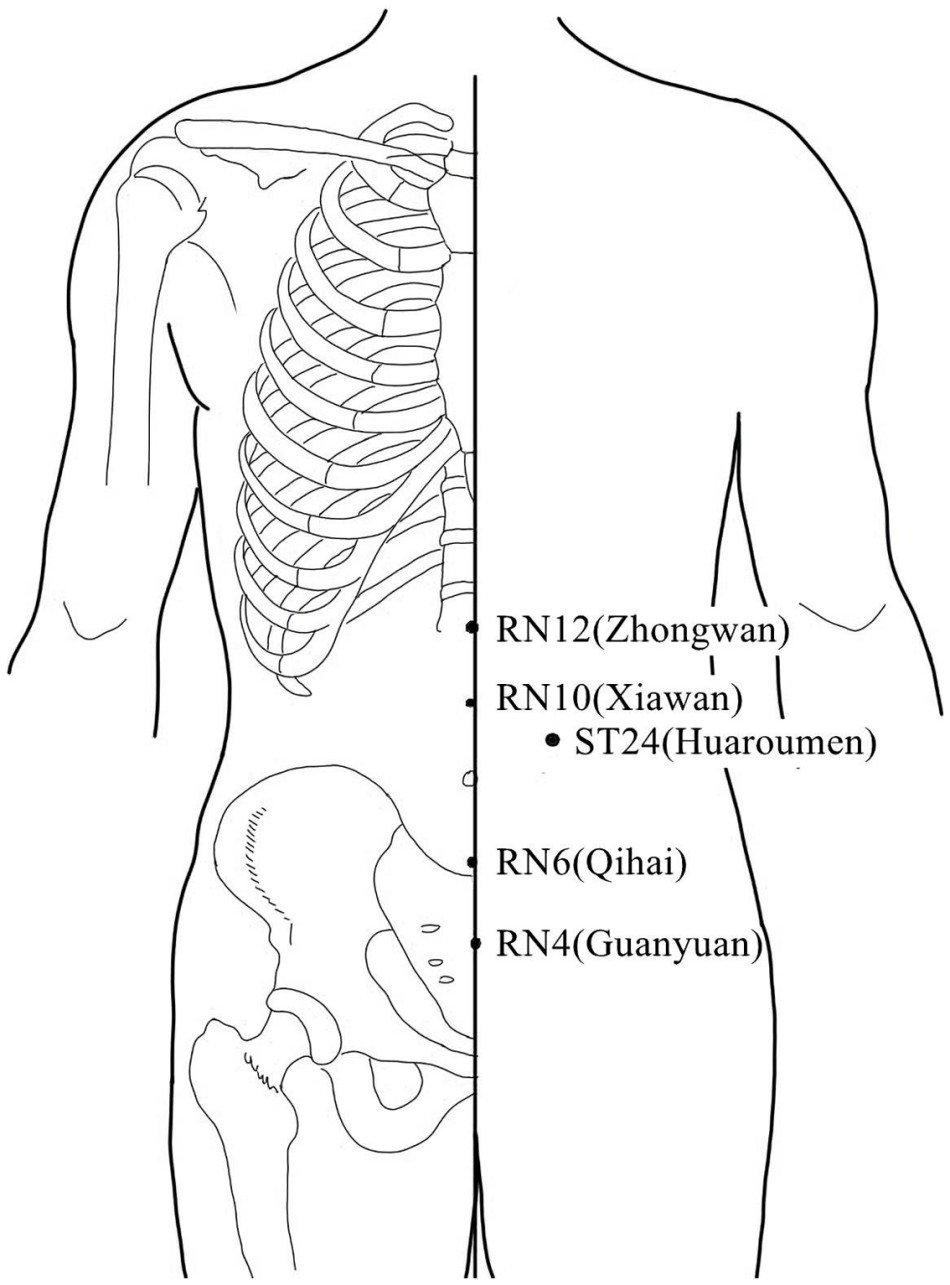
Acupoints in the chest and abdomen.

Acupuncture or EA affects nerve system according to several studies. Researchers showed that low-intensity ES at the ST36 site could activate brainstem vagal efferent neurons or drive catecholamine release from adrenal glands to achieve the target that suppressed systemic inflammation induced by bacterial endotoxins (Liu et al., 2021). Some results suggested that electroacupuncture may be able to modulate extracellular adenosine triphosphate (ATP) levels in the prefrontal cortex of depressive-like maternal separation rats, potentially contributing to its antidepressant effects (Zheng et al., 2023). It is demonstrated that EA pretreatment resulted in increased ambient endocannabinoid (eCB) levels and subsequent activation of ischemic penumbral astroglial cannabinoid type 1 receptors (CB1R) in a middle cerebral artery occlusion (MCAO) model which led to moderate upregulation of extracellular glutamate that protected neurons from cerebral ischemic injury (Yang et al., 2021). Researchers found that EA markedly improved acute lumbar sprain, and EA better improved the rehabilitation and regeneration of force-displacement values and temperature index of the infrared thermogram of the muscle (Fan and Wu, 2015). Acupuncture or EA has been found to have a beneficial clinical effect in treating PNI, according to some researchers. Accelerated nerve regeneration caused by electroacupuncture with intermittent direct current may contribute to the recovery of PNI (Inoue et al., 2011). Some studies demonstrated that short-term acupuncture treatment may result in long-term improvement in mild-to-moderate idiopathic CTS. Acupuncture treatment can be considered an alternative therapy to other conservative treatments for those who do not opt for early surgical decompression (Yang et al., 2011). Some findings indicated that electroacupuncture could improve symptomatology for CTS patients, as evidenced by improved symptomatology, grip strength, electrophysiological function, and physical provocation sign (Ho et al., 2014). The grip strength of CTS patients’ primary symptomatic side dramatically increased following acupuncture treatment indicating an improvement in median nerve motor function obstacle (Mathiowetz et al., 1985). As CTS-induced paresthesias constitute diffuse, synchronized, multidigit symptomatology, researchers found that maladaptive change and correction are consistent with Hebbian plasticity mechanisms. Acupuncture shows promise in inducing beneficial cortical plasticity manifested by more focused digital representations (Napadow et al., 2007b). The acupoints for PNI treatment are listed in Tables 1–5 and shown in Figures 1–5. Table 1 summarizes the evidence of the effect of acupuncture on the repair of peripheral nerve injury.

**Table 1:** Effect of acupuncture on the repair of peripheral nerve injury.

Study	Model	Acupoints	Acupuncture type/parameter	Major effects
Hoang et al. (2012)	Sciatic nerve crush	GB30, GB34	EA/0.8–1 mA, 2 Hz, 15 min	motor recovery↑, target re-innervation pain↓
Ho et al. (2013)	Transected Median Nerve	PC7, PC3	EA/1 mA, 2 Hz, 15 min	Electrophysiological Measurements (Latency, amplitude, MAPs, NCV)↑, Morphologically (axon number, endoneurial area, total nerve area, blood vessel number)↑, Functionally (Grasping Test)↑
Cheng et al. (2001)	Rat sciatic nerve	GB30, GB34	EA/0.8-1 mA, 2 Hz, 15 min	axon density↑, blood vessel area↑, percentage of blood vessel area↑
Li H. et al. (2021)	Upper limb peripheral nerve injury	C6-T1 Jiaji points	EA/once a day, 30 min	Barthel index↑, Fugl-Meyer assessment score↑, motor nerve conduction velocity↑, sensory nerve conduction velocity and amplitude↑, SF-36↑
Napadow et al. (2007a,b)	CTS	Common:TW-5, PC-7 matching aupoint: HT-3, PC-3, SI-4, LI-5, LI-10, LU-5	EA/2 Hz, 10 min	BCTSQ ↑, nerve sensory latencies ↑, grip strength↑, fMRI: inducing beneficial cortical plasticity manifested ↑
Inoue et al. (2011)	Peripheral nerve damage	Anode electrode–proximal to the injured part, cathode electrode–innervated muscle	DCEA/100 Hz, 20 min	functional↑: neurapraxia 2, axonotmesis 3; No functional: axonotmesis 1, neuromesis 1
Ho et al. (2014)	CTS	PC7, PC6	EA/0.8 mA, 2 Hz, 15 min	Elec-Acu group: short clinical questionnaire by Lo and Chiang↑; Acu group: grip strength↑, distal median motor amplitude of the palm-wrist segment↑, Tinel’s sign↓; No changes: two-point discrimination
Yang et al. (2011)	CTS	PC-7, PC-6	MA/30 min	modified GSS↑, DML↑, CMAP↑, MNCV↑, DSL↑, SNAP↑, W-P SNCV↑
Fan and Wu (2015)	Acute lumbar muscle sprain	Bilateral SI-3, Jiaji (EX-B2), Ashi points	EA/10-25 Hz, 20 min	FDVs of the bilateral lumbar muscle↑, temperature index of the lumbar skin↑

MAPs, muscle action potentials; NCV, nerve conduction velocity; CTS, carpal tunnel syndrome; BCTSQ, Boston Carpal Tunnel Syndrome Questionnaire; GSS, Global symptom score; DML, distal motor latency; CMAP, compound muscle action potential; MNCV, motor nerve conduction velocity; DSL, distal sensory latency; SNAP, sensory nerve action potential; W-P SNCV, wrist-palm sensory nerve conduction velocity; FDV, force-displacement value.

**Table 2:** Mechanism of acupuncture on the repair of spinal cord regulation in peripheral nerve injury.

Study	Model	Acupoints	Acupuncture type/parameter	Major effects
Pan et al. (2019)	DPN	BL13, BL20, BL23, LI4, LR3, ST36, SP6	EA/frequency 3 Hz, 20 min	GRP78↓, caspase-12↓, sciatic nerves cell apoptosis↓
Kim et al. (2009)	Capsaicin-induced secondary hyperalgesia	GB30-GB34, BL40-BL60, GV2-GV6, LI3-LI6, SI3-TE8	EA/intensity 3 mA, frequency 2/100 Hz, 30 min	Endogenous spinal mu- and delta-opioid receptors↑
Koo et al. (2008)	Ankle sprain	SI6, LI4	EA/intensity 2 mA, frequency 2/100 Hz, 30 min	Spinal alpha (2)-adrenoceptor↑
Li et al. (2007)	Inflammatory pain	GB30	EA/intensity 3 mA, frequency 10 Hz, 20 min	Supraspinal neurons↑
Mayer et al. (1977)		Ho-Ku’	MA/rotated for 2 min out of 5 min, 30 min	Release of an endogenous substance with narcotic analgesic activity↑

DPN, diabetic peripheral neuropathy; GRP78, glucose-regulated protein 78.

**Table 3:** Mechanism of acupuncture on the regulation of brain regions in peripheral nerve injury.

Study	Acupoint	Intervention and acupuncture type/parameters	Control intervention	Method	Major effects
Matsumoto-Miyazaki et al. (2016)	GV 26, Ex-HN 3, bilateral LI 4, ST 36	MA/10 min	No treatment	TMS	MEP amplitude↑, MEP/Mmax ↑, CMCTs↓, the CST activity of patients with chronic DOC after severe TBI↑
Zhao N. et al. (2018)	At the middle 2/5 of the MS6 line in the affected hemisphere	SA/40 min + LF-rTMS 20 min	SA/40 min	DTI	FMA↑, MBI↑, FAvalue↑, MDvalue↓
Yang et al. (2017)	LI-11, LI-10, TB-5, LI-4, ST-36, GB-34, SP-6, EX-UE9	MA/Deqi, later hold the needle still for 30 min	No stimulation	TMS	Left MEP↓, right MEP↑, IHI↑
Chen et al. (2015)	GB34	MA/1.5 Hz, 1 min baseline-30 s Stimulation-three blocks	Non-acupoint	fMRI	Motor-cognition connectivity↑, compensation of unaffected motor cortex and homolateral synkinesis↓
Napadow et al. (2007a,b)	LI-4	MA/1 Hz, 2 min rest-1 min stimulation-7-min block paradigm	Non-insertive cutaneous stimulation	fMRI	Functional connectivity between the hypothalamus and amygdala: amygdala deactivation↓, hypothalamus activation↑, and vice versa
Wang et al. (2016)	RN12, RN10, RN6, RN4, KL17, ST24, Qipang	Abdominal acupuncture/20 min	Non-insertive cutaneous stimulation	fMRI	MADRS scores↓, SDS scores↓, rsFC between the left amygdala and sgACC/ pgACC↑
Napadow et al. (2005)	ST-36	MA/1 Hz; EA/2 Hz, 100 Hz, 2 min rest-1 min stimulation-7-min block paradigm	Tactile control stimulation	fMRI	Acu, EA: anterior insula hemodynamic signal↑, limbic and paralimbic structures hemodynamic signal ↓ only EA: anterior middle cingulate cortex signal↑, pontine raphe area signal↑
Yan et al. (2005)	Liv3 LI4	MA/1 Hz	Non-acupoints	fMRI	Liv3, LI4: middle temporal gyrus and cerebellum↑, middle frontal gyrus and inferior parietal lobule↓ Liv3: postcentral gyrus, posterior cingulate, parahippocampal gyrus, BA 7, 19 and 41↑, inferior frontal gyrus, anterior cingulate, BA 17 and 18↓ LI4: temporal pole↑, precentral gyrus, superior temporal gyrus, pulvinar and BA 8, 9 and 45↓
Kong et al. (2002)	LI4	EA, MA/3 Hz, 1 min baseline-1 min stimulation-5-min block paradigm		fMRI	EA: precentral gyrus, postcentral gyrus/inferior parietal lobule, and putamen/insula fMRI signal ↑ Acu: posterior cingulate, superior temporal gyrus, putamen/insula fMRI signal↓
Hui et al. (2000)	LI4	MA/120 times per min, 2 min baseline-2 min stimulation-4 min rest-16 min scan time	Tactile stimulation	fMRI	Modulates the activity of the limbic system and subcortical structures
Hui et al. (2005)	ST36	MA/1 Hz, 2 min baseline-2 min stimulation-3 min rest-10 min scan time	Sensory control stimulation	fMRI	An integrated response of the human cerebro-cerebellar and limbic systems to acupuncture stimulation that correlates with the psychophysical response
Li et al. (2015)	SJ5	MA/twirled ± 180°, 60 times per minutes, 30s stimulation-30s rest-6 min scan time		fMRI	The clinical effect of Deqi during acupuncture is based on brain functional changes
Lu et al. (2014)	ST36	MA	Non-point	PET	Bilateral amygdalae activation↑, left temporal lobe activation↑, blood perfusion↑, glycol metabolism↑
Shi et al. (2016)	BL40	MA/depth 2 mm, 5 min	MA/depth 10–20 mm, 5 min	fMRI	Acupuncture modulates the limbic-paralimbic-neocortical network to produce its Deqi effects; The similarity of LPNN and DMN suggests that deep needing may mobilize an important intrinsic brain network for its multiple modulation effects
Wang et al. (2013)	LV3	MA/rotated 180°, 1 Hz, 2 min baseline-2 min stimulation-3 min rest-10 min scan time	Tactile stimulation	fMRI	Pressure was contributing to negative activation of a LPNN; modulatory effects of different needling sensations contribute to acupuncture modulations of LPNN network
Napadow et al. (2009)	PC6	MA/0.5 Hz, 30s stimulation-30s rest-5.5 min scan time	Non-invasive cutaneous stimulation	fMRI	Cognitive load↑, dmPFC activity↑
Fang et al. (2009)	LV3, LV2, ST44	MA/160 times per min, 180°, 1 min stimulation- 1 min rest-6 min scan time	Sham acupoint stimulation	fMRI	Limbic-paralimbic-neocortical system extensive deactivation↓; sensorimotor cortices, thalamus and occasional paralimbic structures activated↑
Dhond et al. (2008)	PC6	MA/twirled ± 180°, 0.5 Hz, 5.5 min baseline-5.5 min stimulation-31.5 min scan time	Non-insertive cutaneous stimulation	fMRI	DMN connectivity↑, SMN connectivity↑, post-stimulation spatial extent of resting brain networks to include anti-nociceptive, memory, and affective brain regions↑
He et al. (2014)	LI4	MA/Deqi, 10 min baseline-10 min stimulation-10 min postacupuncture resting state		fMRI	Connectivity in the primary somatosensory region of both early and late recovery groups↑
Zhang et al. (2016)	LR3, KI3	MA/90–180°, 60–90 times per min, lifted and thrust 0.3–0.5 cm, 30 min	Non-acupoint	fMRI	Number of brain regions with altered brain activity after acupuncture at acupoint combinations↑
Jung et al. (2015)	PC6, HT7	MA/1 Hz	Pseudo-stimulation	fMRI	Salience network↑, default mode network↓
Lee et al. (2013)	ST36	MA/1 min baseline-30s stimulation-30s rest-5 min scan time	Non-acupoint	fMRI	Blood oxygenation level-dependent signal intensity in basal ganglia, limbic system, and cerebellum↓
Napadow et al. (2009)	PC6	MA/0.5 Hz, 30s baseline-30s stimulation-30s rest-5.5 min scan time	Non-insertive cutaneous stimulation	fMRI	Anterior dmPFC activity↑, posterior dmPFC activity↑
Long et al. (2016)	ST36	MA/6 min stimulation-6 min rest-4 min break	Non-acupoint	fMRI	Centrality in parahippocampal gyrus↑, centrality in middle temporal gyrus↑, DMN↑
Lin et al. (2016)	LI4	SNA + MS/twirled rotated, 180°, 1 Hz, 20s for15min	SNA, TNA/15 min; TENS/1 Hz, 20s for 15 min	fMRI	Enhance the acupuncture dose induce different DMN modulatory effects; TNA induces the most extensive DMN modulation
Dhond et al. (2008)	PC6	MA/twirled (∼ ± 180°), 0.5 Hz, 5.5 min rest-5.5 min stimulation-31.5 min scan time	Non-insertive cutaneous stimulation	fMRI	DMN connectivity with pain, affective and memory related brain regions↑, SMN connectivity with pain-related brain regions ↑
Ren et al. (2008)	Neiguan, Waiguan, Sanyinjiao, Zusanli	EA/1 mA, 10 Hz, 30 min			Dendritic spine density↑, ephrin-A5↑, neural plasticity at the peri-infarct cerebral cortex in acute cerebral ischemia rat↑
Liang et al. (2017)	ST36, LI11	EA/2 mA, 1/20 Hz, 30 min			The modified neurologic severity scores↑, neural activity of motor function-related brain regions↑
Wu et al. (2018a,b)	GB30, ST36	EA/0.2 mA, 1/20 Hz, 15 min			Limbic/paralimbic areas fluctuated↑
Maeda et al. (2017)	TW5 PC7 + three additional acupoints	EA/2 Hz, 20 min	Sham acupuncture	fMRI	Primary somatosensory cortex somatotopy↑

MA, manual acupuncture; TMS, transcranial magnetic stimulation; MEP, motor-evoked potential; CMCT, Central motor conduction time; CST, cortico spinal tract; DOC, disorders of consciousness; TBI, traumatic brain injury; DTI, Diffusion tensor imaging; FA, fractional anisotropy; MD, mean diffusion; LF-rTMS, low frequency repetitive transcranial magnetic stimulation; IHI, interhemispheric inhibition; SA, scalp acupuncture; Deqi, a characteristic sensation of aching and tingling; 7-min block paradigm including a 2-min rest, 1-min stimulation, 2-min rest, 1-min stimulation, 1-min rest block; MADRS, Montgomery–Åsberg Depression Rating Scale scores; SDS, Self-Rating Depression Scale scores; sgACC, subgenual anterior cingulate cortex; pgACC, preguenual anterior cingulate cortex; rsFC, resting state functional connectivity; DMN, default mode networks; LPNN, limbic-paralimbic-neocortical network; dmPFC, dorsomedial prefrontal cortex; SMN, sensorimotor network; SNA, single needle acupuncture; TENS, transcutaneous electrical nerve stimulation; TNA, three needle acupuncture; MS, manual stimulation.

**Table 4:** Mechanism of acupuncture on the regulation of factors associated with neuronal response in peripheral nerve injury.

Animal study	Model	Acupoints	Acupuncture type/parameter	Major effects
Liu et al. (2015)	MS	GV6 GV9	EA/1-2 mA, 2/60 Hz, 20 min	NT-3↑, differentiation of oligodendrocyte-like cells from grafted NR-MSCs in the demyelinated spinal cord↑
Hu et al. (2018)	Sciatic nerve injury	GB30 ST36	EA/20 mA, 5 Hz, 15 min	Compare to model-only group: sciatic functional index↑, recovery rate of conduction velocity↑, diameter recovery of the gastrocnemius muscle fibers↑, S100-immunoreactive cells↑, nerve growth factor↑; treatment groups: not differ
Zhao X. et al. (2018)	MCAO	GV20	EA/1-2 mA, 2/10 Hz, 30 min	miR-132↑, SOX2↓
Cui et al. (2017)	SD rats	ST36 SP6	EA/3 mA, 2/15 Hz, 30 min	miR-7a-5p↓, miR-148a-3p ↓, miR-124-3p↑, miR-204-5p ↓, miR-370-3p↓, miR-221-3p ↑, miR-107-3p↑
Du and Liu (2015)	Diabetic models	ST36	EA/1 mA, 10/100 Hz, 30 min	The mRNA and protein level of the enteric neurons↑, GDNF in colon↑, p-Akt in colon↑
Yu et al. (2017)	SNI models	GB30 ST36	EA/2 mA, 5 Hz, 30 min	Agrin ↑, AChR-ε↑, AChR-γ↓
Yu et al. (2017)	Sciatic nerve injury	GB30 ST36	EA/2 mA, 5 Hz, 30 min	SFI↑, tibialis anterior muscle weight↑, muscle fibre CSA↑, agrin expression levels↑, AChR-ε expression levels↑, AChR-γ expression levels↓
Liang et al. (2016)	SD rats	ST36 BL60	EA/1-2 mA, 2/100 Hz, 30 min	PWTs↑, p-p38 MAPK↓, OX-42↓
Shan et al. (2007)	SD rats	GB30 GB34	EA/ranging from 1–2-3 mA, 2/100 Hz, 30 min	Microglial activation↓, MA-induced up-regulation of IL-1β, IL-6, and TNFα mRNA ↓, OX-42-IR↓
Sun et al. (2008)	SD rats	GB30 GB34	EA/1–3 mA, 2/100 Hz, 30 min	Spinal glial activation↓
Ali et al. (2020)	Wistar rats	ST36 SP6	EA/2–3 mA, 2 Hz, 20 min	IL-10↑, β-endorphin↑, neuropathic pain↓
Zhang et al. (2014)	Avulsion injury to the left brachial plexus root	LI11, LI04 ST36, GB34	EA/8 mA, 2–100 Hz, 15–20 min	Mechanical stimulation pain threshold↑, Autotomy scoring↓, β-endorphin↑
Wang et al. (2018)	Sciatic nerve CCI	ST36, GB34 Both ilateral	EA/1 mA, 2/15 Hz, 30 min	Hyperalgesia↓, glial cell activation of the lumbar spinal cord↓, microgliacytes of astrocytes↓, astrocytes activity↓, GFAP protein↓

MS, multiple sclerosis; NT-3, neurotrophin-3; NR-MSCs, neurotrophin-3 and retinoic acid preinduced mesenchymal stem cells; MCAO, middle cerebral artery occlusion; GDNF, glia cell line-derived neurotrophic factor; SNI, sciatic nerve injury; AChR, acetylcholine receptor; SFI, sciatic nerve functional index; CSA, cross-sectional area; PWTs, paw withdrawal thresholds; p38 MAPK, p38 mitogen-activated protein kinase; OX-42, oxycocin-42 (marker of microglia); TNFα, tumor necrosis factor-alpha; IL, interleukin; CCI, chronic constrictive injury; GFAP, glial fibrillary acidic protein.

**Table 5:** Mechanism of acupuncture on the regulation of factors associated with neuronal response in peripheral nerve injury.

Animal study	Model	Acupoints	Acupuncture type/parameter	Major effects
Liu et al. (2015)	MS	GV6 GV9	EA/1-2 mA, 2/60 Hz, 20 min	NT-3↑, differentiation of oligodendrocyte-like cells from grafted NR-MSCs in the demyelinated spinal cord↑
Hu et al. (2018)	Sciatic nerve injury	GB30 ST36	EA/20 mA, 5 Hz, 15 min	Compare to model-only group: sciatic functional index↑, recovery rate of conduction velocity↑, diameter recovery of the gastrocnemius muscle fibers↑, S100-immunoreactive cells↑, nerve growth factor↑; treatment groups: not differ
Zhao X. et al. (2018)	MCAO	GV20	EA/1-2 mA, 2/10 Hz, 30 min	miR-132↑, SOX2↓
Cui et al. (2017)	SD rats	ST36 SP6	EA/3 mA, 2/15 Hz, 30 min	miR-7a-5p↓, miR-148a-3p ↓, miR-124-3p↑, miR-204-5p ↓, miR-370-3p↓, miR-221-3p ↑, miR-107-3p↑
Du and Liu (2015)	Diabetic models	ST36	EA/1 mA, 10/100 Hz, 30 min	The mRNA and protein level of the enteric neurons↑, GDNF in colon↑, p-Akt in colon↑
Yu et al. (2017)	SNI models	GB30 ST36	EA/2 mA, 5 Hz, 30 min	Agrin ↑, AChR-ε↑, AChR-γ↓

MS, multiple sclerosis; NT-3, neurotrophin-3; NR-MSCs, neurotrophin-3 and retinoic acid preinduced mesenchymal stem cells; MCAO, middle cerebral artery occlusion; GDNF, glia cell line-derived neurotrophic factor; SNI, sciatic nerve injury; AChR, acetylcholine receptor; SFI, sciatic nerve functional index; CSA, cross-sectional area.

## 3. Nerve system remodeling-related mechanisms of acupuncture or electroacupuncture for the treatment of peripheral nerve injury

### 3.1. Peripheral nerve fiber regeneration and repair

The primary obstacles to saving neurological function following damage are repair, regeneration, and neuroprotection (Ribeiro et al., 2016; Tallon and Farah, 2017). The potential for regeneration after nerve fiber injury is reflected by the *de novo* expression or upregulation of a massive variety of molecules in the distal nerve fiber tracts (Gordon and Borschel, 2017). So, it is vital to maintain the integrity of the nerve fibers. Electrical stimulation can facilitate peripheral nerve regeneration and target reinnervation (Wenjin et al., 2011; Liu et al., 2013; Zhao et al., 2013). Investigators believe that a weak electric field may affect the regeneration of mammalian peripheral nerves (Schmidt et al., 1997), and extracellular electric fields could influence the orientation of neurite development (Patel and Poo, 1982). Under the influence of a weak electrical field, neurite outgrowth grew more quickly in the direction of the negative pole, but the growth in the direction of the positive pole was suppressed (Song et al., 2004). Studies have discovered that needles used for direct electrical stimulation of the damaged ulnar nerve together with rehabilitation expedite nerve regeneration (Tang et al., 2016). Additionally, electro-needling could increase the number of blood vessels and axon densities in rat peripheral nerves regenerated within a silicone rubber tube (Cheng et al., 2001), after nerve transection, EA could promote the recovery of transected median nerve morphology and function (Ho et al., 2013). According to the experimental results, electroacupuncture can upregulate β-endorphin expression, which reduces neuropathic pain and aid in the healing of damaged brachial plexus nerves (Zhang et al., 2014). According to some research, acupuncture therapy could enhance motor and sensory nerve conduction, scores for the sciatic nerve function index, compound muscle action potential, and motor nerve conduction velocity as well as better promote sciatic nerve regeneration and reduce muscle atrophy while causing less mechanical damage to nerve trunk (Yu et al., 2019). EA was reported to effectively improve functional outcomes following PNI (Zhi et al., 2017). After nerve reconstruction, the regeneration of the motion axis takes a long time (Pagnussat et al., 2012; Sturma et al., 2018). Therefore, EA can both serve as a foundation for nerve regeneration and has the potential to speed up the healing process for damaged nerves.

### 3.2. Mechanism of spinal cord regulation in peripheral nerve injury

#### 3.2.1. Central sensitization

Central sensitization, where the input of noxious stimuli enhances the perceptual response of the central nervous system to pain, is caused by tissue inflammation or peripheral nerve damage and plays an important role in persistent pain (Van Griensven et al., 2020). Previous research has shown that a local cutaneous injury can produce primary hyperalgesia within the injured area and secondary hyperalgesia in the normal surrounding skin, for example, an intradermal injection of capsaicin in humans causes intense pain and hyperalgesia to heat and mechanical stimuli in the surrounding skin. Psychophysical studies in humans supported the conclusions that hyperalgesia was predominantly the secondary type and depended on one set of neurons sensitizing another (“neurogenic hyperalgesia”) and that the latter set of neurons is located in the central and not the peripheral nervous system (Baumann et al., 1991). Central sensitization is accountable for spreading pain and hyperalgesia to uninjured tissue, the process is involved in pain-transmission neurons in the central nervous system (CNS), often in the dorsal horn of the spinal cord (Kim et al., 2009). Second-level sensory neurons, or the relay stations of the conduction of the sensory system, are represented by the neurons of the spinal dorsal horn. The ascending fiber tracts are created by the spinal dorsal horn neurons’ axons as they enter the ipsilateral or contralateral white matter. These ascending fiber tracts then transmit the dorsal root afferents’ nerve impulses to the third-level neurons in the ventroposterolateral thalamic nucleus of the brain, which then transmits them to the cerebral cortex. Some results suggested that acupuncture may simultaneously modulate the resting state functional connectivity (rsFC) of key regions in the descending pain modulation (periaqueductal gray, PAG) and reward systems (ventral tegmental area, VTA), and the amygdala may be a key node linking the two systems to produce antinociceptive effects (Yu et al., 2020). This makes sense because it is widely known that ipsilateral acupuncture has an impact on numerous descending inhibitory networks from the amygdala, periaqueductal gray (PAG), and rostroventral medulla (RVM) which primarily modulate nociceptive signals entering the ipsilateral spinal cord and are influenced by ipsilateral acupuncture (Levine et al., 1991; Van Bockstaele et al., 1991; Manning, 1998; Urban et al., 1999). Acupuncture is demonstrated to activate the ascending sensory pathways, such as the spinal dorsal horn and thalamus, or the descending pain inhibitory mechanisms, such as opioid, adrenergic, and serotonergic pathways, however, the precise areas where EA affects pain are not fully established (Skrabanek, 1984; Tian et al., 2006; Li et al., 2007; Koo et al., 2008).

#### 3.2.2. Neuronal apoptosis

According to previous findings, PNI not only results in localized damage but also trigger the apoptosis of certain spinal cord neurons in corresponding spinal cord segments (Inquimbert et al., 2018). The survival of central neurons plays a key role in determining the effectiveness of peripheral nerve regeneration (Kim and Choi, 2016). After PNI, the corresponding neuron cell bodies will undergo apoptosis because of neurotrophin transmission disorders. And we discovered that glial cell-derived neurotrophic factors and brain-derived neurotrophic factors could alleviate PNI in animal models, and decrease apoptosis and autophagy *in vitro* cell and cell injury model studies (Chou et al., 2014; Kingham et al., 2014). PNI causes apoptosis in spinal motor neurons and sensory spinal ganglion neurons, but it is not extensive and does not affect the entire spinal cord, it is associated with neurons that are connected to the injured peripheral nerves (Ahmad et al., 2015). According to a previous study, acupuncture can ensure and enhance the continuity between peripheral and central nerves, speeding up the process of repairing injured nerves, laying the groundwork for regeneration, and effectively restoring motor conduction (He et al., 2015), and electroacupuncture could dramatically boost facial nerve regeneration by increasing the expression of GDNF and N-cadherin in neurons, preventing neuronal apoptosis (Fei et al., 2019). Some results indicate that EA suppresses spinal nerve ligation (SNL)-induced neuropathic pain by improving neuronal plasticity *via* upregulating the adenosine A2A receptor (A2AR) and the cyclic adenosine monophosphate (cAMP)/protein kinase A (PKA) signaling pathway (Wu et al., 2021a), but more research is still needed to understand the underlying mechanism. Table 2 summarizes the evidence of the mechanism of acupuncture on the repair of spinal cord regulation in peripheral nerve injury.

### 3.3. Synaptic remodeling

The place where neurons join and where information is transmitted is called a synapse. Synapses, which are mostly found on dendritic spines and/or soma, serve a crucial role in the functional connections between neurons and are essential parts of information transmission. The synaptic region contains a variety of enzymes that break down neurotransmitters. Neurotransmitters transmit more slowly and are more degraded when there is a larger synaptic distance. As a result, the structural basis for the remodeling of synaptic transmission function may be the increase in the number of synapses, synaptic vesicle density, and the narrowing of synaptic space. A prior study suggested that the analgesic effect of EA might be connected to the suppression of inflammatory factors and dendritic spine/synaptic remodeling (Wu et al., 2021b).

**Figure 6:**
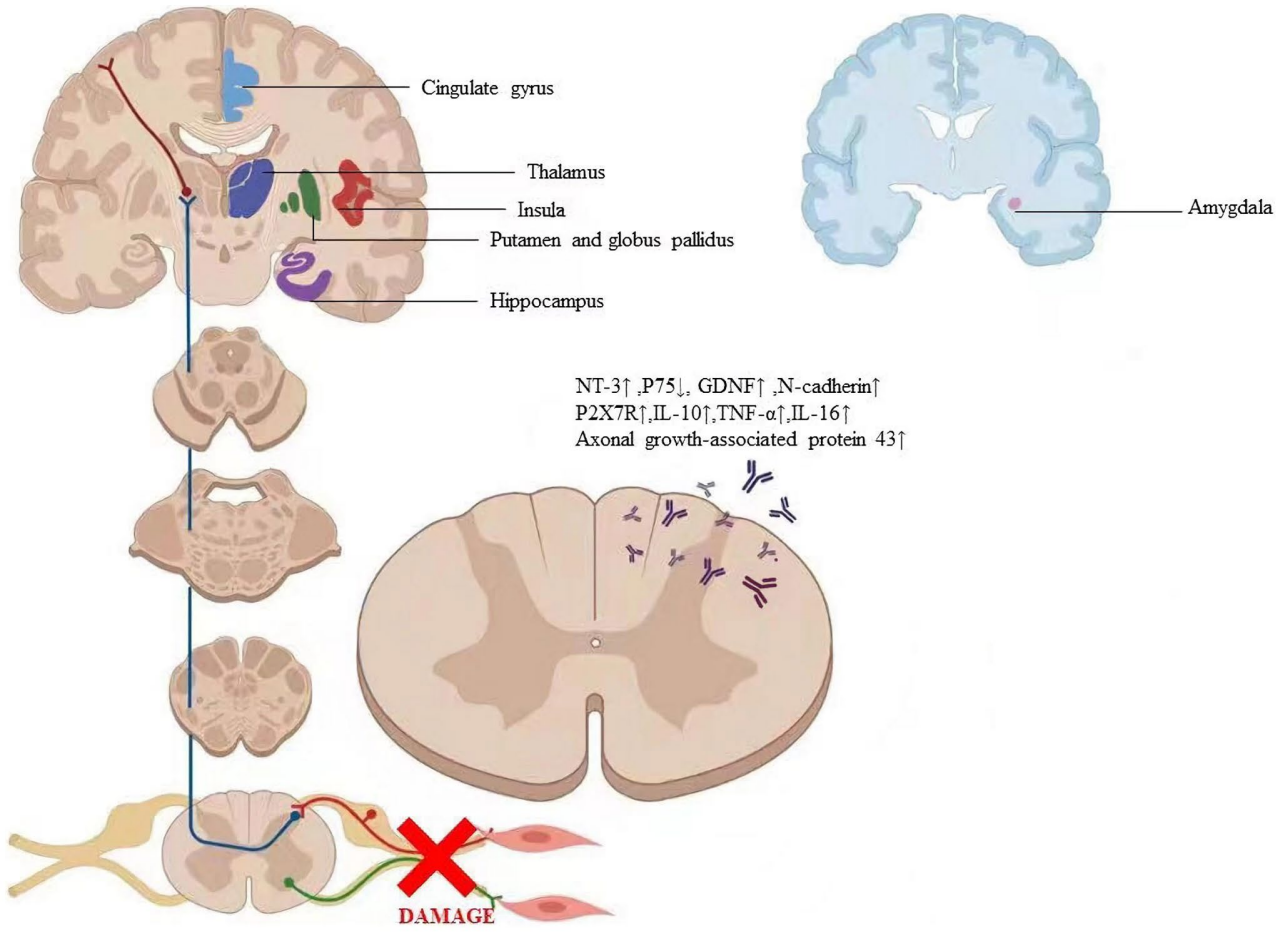
The labeled regions revealed where the particular alterations in the brain are caused by acupuncture. The factors regulated by acupuncture include axonal growth-associated protein 431, NT-31, P751, GDNF1, N-cadherin, P2X7R1, IL-101, TNF-at, and IL-161. Abbreviation: NT-3: Neurotrophins-3, GDNF: Glial cell line-derived neurotrophic factor, P2X7R: P2X7 receptor, IL- 10: Interleukin 10, TNF-α: Tumor necrosis factor-alpha, IL-16: Interleukin 16.

Microglial cells, the smallest glial cells in the nervous system with polysynaptic and plasticity, are the innate immunological effector cells in the central nervous system. Previous research has shown that microglial cells are a crucial target for analgesia by EA (Shan et al., 2007; Sun et al., 2008), EA can reduce neuropathic pain by promoting the production of IL-10 in spinal microglial cells (Ali et al., 2020). EA-induced anti-hyperalgesia may be partially associated with the reduced expression of Phospho-p38 MAPK (p-p38 MAPK) on microglia, and subsequently reducing the activation of oxytocin-42 (OX-42, marker of microglia) in neuropathic pain (Liang et al., 2016). P2X7R overexpression in neuropathic pain models can result in microglia activation and overexpression of TNF-a and IL-1b. Long-term potentiation is avoided and the pain threshold is raised by downregulating P2X7R with P2X7-specific siRNA (Chu et al., 2010; Vadivelu et al., 2015; Shen et al., 2018). Following chronic constriction injury, there was an increase in P2X7R expression in the spinal cord and a corresponding drop in the rat pain threshold (Lin et al., 2018). Therefore, EA may reduce aberrant dendritic spine/synaptic remodeling, and inflammation by downregulating P2X7R to improve neuropathic pain and promote nerve repair (Figure 6; Wu et al., 2021b). Table 3 summarizes the evidence of the mechanism of acupuncture on the regulation of synaptic in peripheral nerve injury.

### 3.4. Regulation of brain regions in peripheral nerve injury

Plasticity following peripheral nerve transection has been demonstrated throughout the neuroaxis in animal models of nerve injury (Fawcett and Keynes, 1990). However, little research has demonstrated the brain changes that occur following peripheral nerve transection and surgical repair in humans. Furthermore, the extent to which peripheral nerve regeneration influences functional and structural brain changes has not been characterized (Taylor et al., 2009). Numerous studies on neural imaging extensively revealed the particular alterations in the brain caused by acupuncture (Figure 6; Kong et al., 2009; Napadow et al., 2009; Lee et al., 2013). Due to the tight relationship between behavioral performance and cortical plasticity, acupuncture may have an impact on cortical plasticity (Klingner et al., 2014; Jung et al., 2015; Zhang et al., 2016). Therefore, we asked whether acupuncture treatment of peripheral nerve injury is accompanied by gray and/or white matter structural changes and whether these changes relate to nerve system remodeling.

#### 3.4.1. The endogenous opioid peptide system network

Numerous brain regions and nuclei, including the caudate nucleus, septal area, arcuate nucleus, periaqueductal gray, and nucleus raphe magnus, all of which contain opioid peptides, and μ, δ, and κ receptors, are involved in the transmission of acupuncture signals (Zhao, 2008). The hypothalamic arcuate nucleus is a significant structure in the endogenous opioid peptide system and a crucial region that mediates low-frequency electroanalgesia (Takeshige et al., 1991). Researchers found that acupuncture at HT7 points attenuates behavioral manifestation of alcohol dependence by activating endorphinergic input to the nucleus accumbens (NAc) from the arcuate nucleus(ARC) (Chang et al., 2019). The arcuate nucleus may control electroanalgesia based on the fact that its axons branch out to the adjacent nucleus accumbens, septum, periaqueductal gray, and locus coeruleus. In praxiology and electrophysiology, activating the arcuate nucleus will boost analgesic effects, increased the activity of neurons in the dorsal raphe nucleus and decreased the activity of neurons in the locus coeruleus (Kwon et al., 2004; Yoon et al., 2013). According to studies, more genes were differentially regulated by low-frequency EA than high-frequency EA (154 vs. 66 regulated genes/ESTs) in Arc, especially those related to neurogenesis (Wang et al., 2012). These findings suggest that the opioid-system network that includes the dorsal horn, periaqueductal gray, nucleus raphe magnus, and the arcuate nucleus is crucial for electroanalgesia (Mayer et al., 1977; Han et al., 1991).

#### 3.4.2. Cortical remodeling

##### 3.4.2.1. Remodeling pattern of areas of sensorimotor integration

Maintaining synergy and appropriate sensory and motor function depends on the sensorimotor circuit’s integrity (Yao et al., 2022). PNI disrupts the sensorimotor circuit’s integrity and results in the loss of sensory and motor feedback, which may result in changes to the sensory-motor network’s intrinsic activity (Taylor et al., 2009). However, the adult brain is capable of profound plasticity (Li C. et al., 2021). The fMRI study showed that insertion of the acupuncture needle at acupoint ST 36 significantly affected the proprioceptive brain activation by decreasing blood oxygenation level-dependent signal intensity in basal ganglia, limbic system, and cerebellum (Lee et al., 2013), and EA could reduce chronic pain, so it can be used to treat the paresthesia brought on by PNI persisted (Lu et al., 2021). Current research showed that acupuncture at local versus distal sites may improve median nerve function at the wrist by somatotopically distinct neuroplasticity in the primary somatosensory cortex following therapy (Maeda et al., 2017). Some studies found that after sciatic nerve injury, local synaptic activity increased in the ipsilateral somatosensory cortex and decreased in the contralateral somatosensory cortex (Zuo et al., 2010; Nugent et al., 2015), and the plasticity changes following PNI were situated in homologous regions of the ipsilateral hemisphere in addition to the contralateral hemisphere (Fornander et al., 2016). Acupuncture at GB34 may increase motor-cognition connectivity meanwhile decrease compensation of unaffected motor cortex and homolateral synkinesis (Chen et al., 2015). EA at ST 36 and LI 11 could enhance the neural activity of motor function-related brain regions, including the motor cortex, dorsal thalamus, and striatum in rats (Liang et al., 2017). The results of resting-state functional MRI connectivity show that acupuncture induces significant connectivity changes in the primary somatosensory region of recovery patients with Bell’s palsy (He et al., 2014). Acupuncture increased sensorimotor network (SMN) connectivity with pain-related brain regions (ACC, cerebellum) (Dhond et al., 2008). Nervous damage causes the sensorimotor circuit to lose its integrity, which may prompt the brain to reorganize itself to make up for the damaged sensorimotor circuit. However, these alterations in the brain could be beneficial or harmful (Mohan and Vanneste, 2017). Although EA treatment might reverse the maladaptive cortical plasticity, the status of the brain has not recovered to a level consistent with normalcy (Wu et al., 2018a).

##### 3.4.2.2. Pain network of brain region

It has been demonstrated in both a human and a rat model that nerve transection in PNI typically causes paresthesia or pain before reinnervation (Campbell and Meyer, 2006; Gu et al., 2016; Peng et al., 2016; Lim et al., 2017). Researchers found that the activity pattern of the region associated with pain was remarkably coordinated (Lefaucheur et al., 2009). The changes in regions of the brain, such as the thalamus, amygdala, and cingulate, involved in pain modulation were observed under EA stimulation. For Carpal Tunnel Syndrome patients responding to acupuncture, functional connectivity was found between the hypothalamus and amygdala--the less deactivation in the amygdala, the greater the activation in the hypothalamus, and vice versa. Furthermore, hypothalamic response correlated positively with the degree of maladaptive cortical plasticity in Carpal Tunnel Syndrome patients (inter-digit separation distance; Napadow et al., 2007a). The thalamus, which relays sensory data to the cerebral cortex and is the most significant sensory integration region, has a strong correlation with pain (Çetin et al., 2014; Zitnik et al., 2014). It is well known that the amygdala, hippocampus, and parahippocampus are crucial for regulating emotions, motivation, memory, and the affective aspect of pain (LeDoux, 2000; Zubieta et al., 2001; Mears and Pollard, 2016). Some findings demonstrate the additive effect of acupuncture on antidepressant treatment and suggest that this effect may be achieved through the amygdala and the anterior cingulate cortex (ACC) (Wang et al., 2016). Researchers found higher centrality in the parahippocampal gyrus and middle temporal gyrus after ST36 stimulation. These regions are positively correlated to major hubs of the default mode network, which might be the primary network affected by chronic pain (Long et al., 2016). Studies showed that conventional methods to enhance the acupuncture dose induce different DMN modulatory effects. Conventional methods of enhancing the acupuncture dose could potentially be applied as a means of modulating brain activity (Lin et al., 2016).

##### 3.4.2.3. Limbic-paralimbic system

A group of brain regions known as the limbic system have been linked to emotion, memory, and sensation. Numerous earlier research showed that acupuncture not only stimulated sensorimotor regions of the brain but also deactivated extensive regions of the brain, including the limbic-paralimbic system (Fang et al., 2009; Napadow et al., 2009; Chae et al., 2013). The “Deqi” sensation has been described as a phenomenon of concerted attenuation of signal strength in the para-limbic regions through fMRI investigations. Additionally, acupuncture’s “Deqi” effects may be mediated by modulation of the limbic-paralimbic-neocortical network (Hui et al., 2005; Shi et al., 2016). Acupuncture’s effects on depression, schizophrenia, ischemic stroke, and Alzheimer’s disease may be brought on by the limbic system, according to some animal and clinical investigations (Lu et al., 2014; Bosch et al., 2015; Li et al., 2015; Wang et al., 2016). Researchers discovered that, while treating PNI following surgical repair, the brain plasticity generated by longitudinal EA intervention also occurred in limbic/paralimbic areas. The fMRI experiments of manual acupuncture at LI4, ST36, and LV3 showed the weakness of signal in several limbic, paralimbic, and neocortical areas (Kong et al., 2002; Hui et al., 2005; Napadow et al., 2005; Yan et al., 2005). Some hypothesize that voluntary slow deep breathing functionally resets the autonomic nervous system through stretch-induced inhibitory signals and hyperpolarization currents propagated through both neural and non-neural tissue which synchronizes neural elements in the heart, lungs, limbic system, and cortex (Jerath et al., 2006). The anterior cingulate cortex and nearby medial prefrontal cortex, insula, hippocampus, amygdala, and midcingulate cortices were consistently linked to peripheral markers of autonomic nervous system activity, according to recent meta-analyzes and other reviews (Thayer et al., 2012; Beissner et al., 2013; Gianaros and Wager, 2015; Myers, 2017). According to the hypothesis, the limbic system’s fluctuating behavior was caused by a conflict between the up-regulating effects of paresthesia or pain and the down-regulating effects of EA intervention (Beissner and Henke, 2011). Previous research suggested that acupuncture could promote the regeneration of peripheral nerves (He et al., 2015). It demonstrated the changes in the brain caused by the long-term therapeutic effects of EA, which were manifested as a synchronized pattern of activation in the somatosensory and pain-related areas, as well as a fluctuating pattern in the limbic/paralimbic system (Wu et al., 2018b).

#### 3.4.3. White matter remodeling

According to some studies, corticospinal tract(CST) and spinothalamic tract (STT) fibers are similarly altered when peripheral nerve injury occurs (Zhang et al., 2019). Previous research demonstrated that neural pathway injuries, such as brain and spinal cord injuries, caused STT fibers to adapt or be maladapt (Wang and Thompson, 2008; Wasner et al., 2008). According to findings from a rat study, chronic pain stimuli of the sciatic nerve could lead to the hyperexcitability of ventral posterior thalamus neurons, which were the major termination of STT fibers and responsible for the signal relay (Miki et al., 2000). Neuronal hyperexcitability could be an inappropriate reaction to a reduction or absence of sensory input. Although there was little evidence that acupuncture improved sensory function, some researchers thought that the plasticity alteration in STT may also be predicted to restore sensory function. Previous studies found that acupuncture treatment has a complex effect on the corticospinal system for a variety of disorders (Chen et al., 2015; Yang et al., 2017). It is demonstrated that based on routine rehabilitation treatment, scalp acupuncture plus low-frequency rTMS can promote white matter tracts repair better than scalp acupuncture alone for stroke hemiplegic patients (Zhao N. et al., 2018). Prior research changed the focus of plasticity from gray matter, which includes the motor cortex, to white matter, which includes the CST. Cortical alterations were anticipated to lead to modifications in efferent neural pathways (Matsumoto-Miyazaki et al., 2016). Acupuncture also aided in the recovery process and ameliorated the efferent neural pathway’s longitudinal prognosis (Zou et al., 2019). Table 4 summarizes the evidence of the mechanism of acupuncture on the regulation of brain regions in peripheral nerve injury.

### 3.5. Regulation of neurotrophic factor associated with PNI by acupuncture

Peripheral nerves in mammals can regenerate after being damaged (Menorca et al., 2013; Scheib and Höke, 2013). Schwann cell proliferation is frequently thought to be related to the mechanism behind peripheral nerve regeneration. In particular, neurotrophic substances released by Schwann cells are crucial for the regeneration of peripheral nerves (Jessen et al., 2015). According to research, neurotrophic factors secreted by Schwann cells as well as extracellular matrix and cell adhesion molecules can induce, stimulate, and modulate axon regeneration and the development of myelin sheaths (Madduri and Gander, 2010). Based on this discovery, a proposal was put forth that the distal portions of the nerve guided the regeneration of peripheral nerves, helping to extend neurites and restore innervation through identification and communication between neurons and neurogliocytes. After the sciatic nerve cut off, some researchers found that Schwann cells produced S100, suggesting central nervous system damage. For peripheral nerve injury, low-frequency EA (5 Hz) had the better effect, and the results were most pronounced in the early stages (Cheng et al., 2014; Liao et al., 2017). Neurotrophic factors, cell adhesion molecules (including L1, NCAM, and N-cadherin), transcription factors, growth-stimulating agents, extracellular matrix components, intracellular signaling enzymes, and proteins regulating cell-surface cytoskeletal interactions are related to neuronal response (Figure 6).

#### 3.5.1. Neurotrophic factors

Nerve growth factor (NGF) is the earliest discovered neurotrophic factor and can nourish neurons and promote neurite sprouting (Yu et al., 2014).In response to nerve damage, NGF expression was upregulated, creating the ideal conditions for axon regeneration, the restoration of the connection between axons and Schwann cells, and the reinnervation of target locations to enhance nerve growth (Tang et al., 2013). After peripheral nerve damage, EA may strengthen new axonal connections, which would ultimately accelerate the transfer of NGF. Additionally, EA was able to promote the growth of Schwann cells, which greatly increased the amount of NGF released (Hu et al., 2018).

In the peripheral nervous system, Schwann cells (SCs) are the predominant glial cells. Following PNI, SC migration and proliferation result in the release of a variety of neurotrophic factors (NTFs), which are beneficial for preserving the survival of injured neurons, encouraging nerve fiber regeneration, and fostering the formation of new synapses (Dai et al., 2013). It has been demonstrated that EA may promote the recovery of neuroplasticity in rats subjected to spinal cord transection. This could be attributed to the systematic regulation of NTFs and their receptors after EA.EA stimulation at ST36 in rats increases the expression of axonal growth-associated protein 43 in neurons of the dorsal root ganglia, promoting axonal development (Wang et al., 2017). EA may alleviate tibialis anterior muscle atrophy induced by sciatic nerve injection injury by upregulating agrin and AChR-ε and downregulating AChR-γ (Yu et al., 2017). Some studies found that EA increased NT-3 levels and promoted differentiation of oligodendrocyte-like cells from grafted NR-MSCs in the demyelinated spinal cord and induced MSC transplantation combined with EA treatment not only increased MSC differentiation into oligodendrocyte-like cells forming myelin sheaths but also promoted remyelination and functional improvement of nerve conduction in the demyelinated spinal cord (Liu et al., 2015). Some researchers indicate that electroacupuncture and moxibustion promoted nerve regeneration and functional recovery, which mechanism might be associated with the enhancement of Schwann cell proliferation and upregulation of nerve growth factor (Hu et al., 2018).

As one of the most significant NTFs, persistent exogenous BDNF release in the damaged peripheral nerve may be a helpful tool to boost axonal density (Lopes et al., 2017). In the recovery process after peripheral nerve injury in rats, electrical muscle stimulation increases intramuscular BDNF levels (Willand et al., 2016). Studies have shown that BDNF actively contributes to the regeneration of motor neurons and the restoration of motor function. The amount of the protein BDNF, which has been identified as a crucial regulator of axon regeneration, was significantly enhanced in the spinal cord and DRG following PNI (Santos et al., 2016; Sanna et al., 2017; McGregor and English, 2018).

Previous research demonstrated that glial cell-derived neurotrophic factor (GDNF) was the most effective survival factor described for motoneurons *in vitro* (Höke et al., 2002). Recent investigations have proven GDNF’s function in neuronal protection and axonal regeneration *in vivo* (Allen et al., 2013; Kopra et al., 2015). Researchers discovered that GDNF mRNA is expressed in the cytoplasm membrane, notably in the neuromuscular junctions, and less in the axons and Schwann cells (Suzuki et al., 1998). To protect neurons, GDNF binds to N-cadherin and initiates the intracellular PI3K/Akt signaling pathway (Wang et al., 2014). Researchers found that EA with high-frequency and long-term stimuli at acupoint ST-36 can induce regeneration of lost enteric neurons in diabetic rats, and GDNF and PI3K/Akt signal pathway may play an important role in EA-induced regeneration of impaired enteric neurons (Du and Liu, 2015).

#### 3.5.2. N-cadherin

Cadherins tend to form dimers or multimers, and the extracellular peptide chain partially folds to form five or six repeat domains (Stevens et al., 2023). Calcium ions are bound between the repeat domains, thus imparting rigidity and strength to the cadherin molecule (Oroz et al., 2011). The more calcium ions are bound, the more rigid the cadherin is, so when calcium ions are removed, the rigidity of the extracellular part of the cadherin is lost. Researchers revealed that electroacupuncture may encourage nerve regeneration through an increase in calcium concentration, which activates neuron outgrowth and repair (McCaig et al., 2002). Similarly to NCAM and integrin 1, N-cadherin is a calcium-dependent neuronal cell surface protein that facilitates adhesion and signal transduction (Neugebauer et al., 1988). Ret has a cadherin-like domain in its extracellular region that interacts with the GDNF/GDNF family receptor α1 (Oppenheim et al., 1995; Kjaer and Ibáñez, 2003). Some studies have confirmed that electroacupuncture promotes regeneration of peripheral facial nerve injury in rabbits, inhibits neuronal apoptosis, and reduces peripheral inflammatory response, resulting in the recovery of facial muscle function, which is achieved by up-regulating the expression of GDNF and N-cadherin in central facial neurons (Fei et al., 2019). GDNF binds to N-cadherin, a transmembrane cell adhesion molecule, to exercise its neuroprotective impact on dopaminergic neurons (Cao et al., 2010; Patil et al., 2011; Zuo et al., 2013).

#### 3.5.3. MicroRNAs

MicroRNAs (miRNAs), which comprise between 2 and 3 percent of all human genes and primarily function to silence genes by binding to people’s targets’ 3′ untranslated regions (3′ UTR), are a vital and significant component of the regulation over a variety of signaling pathways and biological functions in the restoration of PNI (Wanke et al., 2018; Treiber et al., 2019), so MiRNAs have gained growing interest in the field of peripheral nerve regeneration (Yu et al., 2015). Numerous studies have shown that miRNAs play a key role in the recovery process after PNI. For instance, miRNA expression in rat dorsal root ganglia (DRG) tissues was drastically altered following sciatic nerve damage (Li et al., 2012). To increase rat DRG neurons’ survival and prevent apoptosis following sciatic nerve damage, miR142-3p can target CDKN1B and TIMP3. MiR-7 influences repair following PNI by targeting cdc42 and preventing neural stem cell migration and proliferation. By targeting GF1-A-binding protein 1, miR-221-3p may prevent SCs from maturing into myelin sheaths when they are cocultured with DRG neurons (Nab1) (Zhao L. et al., 2018; Zhou et al., 2018). Researchers have demonstrated miR-1-targeting BDNF in the regulation of SC proliferation and migration following nerve injury; in addition, miR-1 modulated chronic neuropathic pain in rats by targeting cx43 and BDNF, and miR-206 can target BDNF to the regulation of the MERK-ERK signaling pathway to influence neuro stress pain (Neumann et al., 2015; Yi et al., 2016; Sun et al., 2017). Liu et al. (2018) have discovered that miR-1b overexpression inhibited RSC96’s migration and proliferation while boosting cell apoptosis. Similar targets and expression profiles are used to identify miRNAs that belong to the same family. Numerous miRNAs, including miR-129 and miR-195, had altered expression following PNI, according to studies (Shi et al., 2013; Zhu et al., 2018). Using miR-132 to target SOX2-mediated axonal regeneration, it has been found that EA improved neurobehavioral functional recovery after ischemic stroke (Zhao X. et al., 2018). According to another study, let-7b-5p, miR-148a-3p, miR-124-3p, miR-107-3p, and miR-370-3p were confirmed to participate in EA tolerance probably through the functional categories related to nerve impulse transmission, receptor signal pathways, and gene expression regulation, as well as pathways related to MAPK, neurotrophin, fatty acid metabolism, lysosome, and the degradation of valine, leucine, and isoleucine (Cui et al., 2017). MiR-1b expression was initially found to be highly downregulated in the local nerve following EA in a prior investigation, and EA may promote the proliferation, migration of SC, and nerve repair after PNI by regulating miR-1b, which targets BDNF (Liu et al., 2020). Table 5 summarizes the evidence of the mechanism of acupuncture on the regulation of factors associated with neuronal response in peripheral nerve injury.

## 4. Conclusion and consideration

With in-depth research on the mechanism underlying the effects of acupuncture in treating PNI, we now fully appreciate the importance of nerve system remodeling in this process. we have found that after peripheral nerve injury, not only is the injury limited to the local area of the nerve, but the entire nervous system is altered accordingly. Acupuncture not only reverses the poor remodeling of the nervous system but also activates the central opioid system and the innate immunological effector cells in the nervous system to ameliorate pain. Acupuncture can promote the proliferation and migration of Schwann cells and the release of neurotrophic substances such as nerve growth factors, accelerating the regeneration and repair of nerve fibers. Based on the view that acupuncture affects the remodeling of the nervous system, it is believed that in the treatment of peripheral nerve injury, acupuncture should not only be applied to the local acupoint of peripheral nerve injury but also be combined with scalp acupuncture and Jiaji acupoints. At present, there are many studies on the mechanism of acupuncture affecting nerve remodeling, but there are still many limitations. First, the reduction of inflammatory pain by acupuncture may be related to the interaction between glial cells and neurons in the spinal cord, but the mechanisms of action of microglia and astrocytes after acupuncture are different. It is not known which factors influence their activation timing and molecular signaling within the cell. Secondly, in the process of acupuncture treatment of peripheral nerve injury, the related neural circuit, functional area, and network connection at the central nervous system level are still not clear. Thirdly, it is known that neurotrophic factors are involved in the repair process of peripheral nerve injury, but the production site, production time, and transport route of neurotrophic factors during acupuncture treatment need to be further improved. Finally, current animal models have drawbacks because they show an acute inflammatory response and short-lived hyperalgesia after PNI, both of which become attenuated over time. Most studies have only examined the protective effect of acupuncture in the initial phase of PNI. Better models or clinical trials are needed to explore the effectiveness of acupuncture in the process of chronic nerve repair. Overall, this review of studies provides strong evidence for the usefulness of acupuncture in the treatment of PNI. The elucidation of the mechanisms underlying the effects of acupuncture in the treatment of PNI from the perspective of nervous system remodeling will open a variety of opportunities for further applications of acupuncture and a combination of acupuncture and drugs for treating, managing, and accelerating nerve repair. Therefore, the continuation of research on this topic is extremely important.

## Author contributions

YY: Conceptualization, Data curation, Formal analysis, Methodology, Writing – original draft, Writing – review & editing. CR: Writing – review & editing. TY: Writing – review & editing. SW: Writing – review & editing. HS: Conceptualization, Methodology, Writing – review & editing. XY: Methodology, Writing – review & editing. LZ: Funding acquisition, Writing – review & editing. XM: Writing – review & editing. WG: Writing – review & editing. YD: Writing – review & editing. FH: Funding acquisition, Supervision, Validation, Writing – review & editing.
